# Quality of Life for Women with Human Papillomavirus-induced Lesions

**DOI:** 10.1055/s-0040-1709192

**Published:** 2020-04

**Authors:** Natália Maria Vieira Pereira-Caldeira, Fernanda Garcia Bezerra Góes, Maria Cristina Mendes de Almeida-Cruz, Juliano de Souza Caliari, Fernanda Maria Vieira Pereira-Ávila, Elucir Gir

**Affiliations:** 1 Department of General and Specialized Nursing, Programa Enfermagem Fundamental, Escola de Enfermagem de Ribeirão Preto, Universidade de São Paulo, Ribeirão Preto, SP, Brazil; 2 Department of Nursing of Rio das Ostras, Universidade Federal Fluminense, Rio das Ostras, RJ, Brazil; 3 Department of Nursing, Instituto Federal de Educação Ciência e Tecnologia do Sudeste de Minas Gerais, Passos, MG, Brazil

**Keywords:** papillomavirus infections, quality of life, women's health, gynecology, qualitative research, infecções por papillomavirus, qualidade de vida, saúde da mulher, ginecologia, pesquisa qualitativa

## Abstract

**Objective** To reveal the changes in the quality of life reported by women with Human papillomavirus (HPV)-induced lesions.

**Methods** This is a cross-sectional, descriptive-exploratory study of a qualitative approach performed from June to August 2016. Semi-structured face-to-face interviews based on five questions on the concept of quality of life were used. The data were submitted to thematic analysis. All ethical aspects have been contemplated.

**Results** A total of 20 women aged between 25 and 59 years old were interviewed. From the analysis of the data, the following thematic units emerged: physical and emotional changes, especially complaints of pruritus, discharge and pain, worry, fear, shame and sadness; changes in sexual and affective relationships with decreased libido, dyspareunia and interruption of sexual activity; changes in social relationships resulting in absenteeism at work.

**Conclusion** Human papillomavirus infection impairs the quality of life of women as it significantly affects sexual, affective, physical, emotional, and everyday habits. Therefore, HPV infection can lead to exponential changes in the quality of life of women, which can be mitigated by the availability of sources of support such as family, friends and the multi-professional team, helping to improve knowledge and cope with HPV.

## Resumo

**Objetivo** Desvelar as alterações na qualidade de vida referidas por mulheres com lesões induzidas pelo papilomavírus humano (HPV).

**Métodos** Trata-se de um estudo transversal, descritivo-exploratório, de abordagem qualitativa, realizada no período de junho a agosto de 2016. Foram utilizadas entrevistas semiestruturadas face a face, a partir de cinco questões fundamentadas no conceito de qualidade de vida. Os dados obtidos foram submetidos a análise temática. Todos os aspectos éticos foram contemplados.

**Resultados** Foram entrevistadas 20 mulheres com idades entre 25 e 59 anos. A partir da análise dos dados, emergiram as seguintes unidades temáticas: alterações físicas e emocionais com destaque para as queixas de prurido, corrimento e dor, preocupação, medo, vergonha e tristeza; alterações nas relações sexuais e afetivas com diminuição da libido, dispareunia e interrupção da atividade sexual; alterações nas relações sociais com ausências consecutivas no trabalho.

**Conclusão** A infecção pelo HPV prejudica a qualidade de vida das mulheres, uma vez que afeta de maneira considerável os aspectos sexuais, afetivos, físicos, emocionais e hábitos cotidianos. Portanto, a infecção pelo HPV pode acarretar mudanças exponenciais na qualidade de vida de mulheres, as quais podem ser amenizadas pela disponibilidade de fontes de apoio, como família, amigos e equipe multiprofissional, que auxiliam no nível de conhecimento e enfrentamento do HPV.

## Introduction

Human papillomavirus (HPV) is a group of 226 viruses cataloged by the International Human Papillomavirus (HPV) Reference Center, in which each type of this large group receives a number. Only 13 types are evaluated as oncogenic because they present a greater probability or risk of generating persistent infections and precursor lesions. They usually do not have symptoms and are spontaneously eliminated by the organism.^1^ ^2^ ^3^

The Information Center on HPV and Cervical Cancer (ICO) estimated that there were ∼ 5,880,000 HPV-infected people in the world in 2017 and 2,784,000 women > 15 years old.^4^ In Brazil, an epidemiological study on the prevalence of HPV infection indicated the overall rate of 54.6% of infected people between 16 and 25 years old.^5^

Although HPV has the ability to infect the epithelium of both genders, the damage done to women is greater and more frequent. According to the Centers for Disease Control and Prevention (CDC), HPV-related cancers are not commonly found in men, with HPV infection being the most common sexually transmitted infection (STI).^3^ In addition, women have different biological characteristics to men, with less thinner genital mucosa with a larger contact surface, favoring a higher risk of contagion.^6^

The social and economic inequalities experienced by women with HPV infection have several consequences related to stigma and prejudice, impacting their family, social, affective and sexual relationships.^7^ In today's society, women play a variety of roles like mother, wife, daughter, and housewife, and sometimes they neglect to look after themselves, with a negative effect on their quality of life.

The World Health Organization (WHO) Quality of Life Group defines quality of life as *“the individual's perception of their position in life in the culture and value system context in which they live and in relation to their goals, expectations, standards, and concerns.”* 8

Therefore, evaluating the quality of life of women with HPV infection is an important tool for the development of interventions regarding their physical, psychosocial and economic impact.^9^ ^10^

In addition, the broad concept of quality of life proposed by Fayers et al^11^ includes important aspects that include physical, emotional, cognitive and sexual functioning, physical symptoms and toxicity, social well-being, existential issues, and also the general state of health. Therefore, the aim of the present study was to reveal the changes in quality of life reported by women with human papillomavirus-induced lesions.

## Methods

This is a cross-sectional, descriptive-exploratory, qualitative study performed from June to August 2016 in an outpatient clinic of a public hospital in the interior of the state of São Paulo, Brazil, specializing in infectious diseases in gynecology.

To obtain a heterogeneous sample, the participants in the study were women aged ≥ 18 years old with a proven diagnosis of HPV infection with clinical lesions (condylomatosis) and subclinical lesions. The diagnosis was made through clinical examination and biopsies with histopathological examination. In the cases of subclinical lesions of cervical intraepithelial neoplasia II and III, the diagnosis was made through pap smears (cytopathology), colposcopy and biopsies. For subclinical lesions, vulvar intraepithelial neoplasia and vaginal intraepithelial neoplasia, the diagnosis was made through clinical examination and biopsies with a histopathological examination.

The determination of the presence of the viral DNA HPV in the samples of biopsies collected was performed through the molecular diagnostic technique with the polymerase chain reaction (PCR) method. Polymerase chain reaction is one of the most sensitive and commonly used techniques for detecting HPV.^12^ ^13^ ^14^ It is a molecular biology method based on the selective amplification of a specific DNA sequence. Polymerase chain reaction-amplified products can be analyzed in several ways, including gel electrophoresis (agarose or polyacrylamide), dot blotting, reverse-blot hybridization, and direct sequencing of DNA.^15^

Those with coinfection by the human immunodeficiency virus, considered a confounding factor, were excluded. The selection of the women was a matter of convenience, selecting those who came to the clinic on the day of the scheduled medical appointment.

To evaluate the quality of life, the data were collected through a semistructured face-to-face interview, based on five questions proposed by Fayers et al^11^ on the concept of quality of life.

The questions were: 1: Do you know why HPV infection happens? 2: Has there been any change in your sex life after the diagnosis of HPV?; 3: Has there been any change in your daily life (work, daily activities) after the diagnosis of HPV?; 4: How do you feel about the diagnosis of HPV?; 5: Do you feel any physical discomfort related to HPV?

The interviews lasted from 30 to 40 minutes. Nobody refused to participate, and none of the participants dropped out during data collection or after. The interviews were conducted in a private setting in the outpatient clinic, where only the researcher and the interviewee were present, to preserve the privacy of the participants.

The number of participants was based on the precepts of theoretical data saturation, as the interviews revealed the recurrence of ideas and practices related to the object of study.^16^

For the analysis of the data, the fully transcribed interviews generated a textual corpus that was submitted to thematic analysis after an exhaustive reading of the reports.^16^

An alphanumeric code made up of the letter “E” and the arabic numeral corresponding to the sequence of the interviews was used for the identification of the participants interviewed, thereby ensuring their anonymity.

All of the recommendations of Resolution 466/12 of the National Health Council were respected; therefore, the interviews were only conducted after approval of the study by the Research Ethics Committee (CAAE: 38995114.0.0000.5393, opinion n°1,080/473), and all of the participants read and signed the Informed Consent Term.

## Results

A total of women aged between 25 and 59 years old were interviewed. Their cultural, social and educational characteristics are described in Table 1.

**Table 1:** Distribution of women (*n* = 20) according to cultural, social and educational characteristics

Variables	*n* (%)
Ethnicity / skin color (self-declared)	
White	7(35.0)
Brown	11(55.0)
Black	2(10.0)
Occupation	
Housekeeper	10(50.0)
Saleswoman	4(20.0)
Unemployed	2(10.0)
Merchant	2(10.0)
Retired	2(10.0)
Schooling (years studied)	
9–12	17(85.0)
> 12	3(15.0)
Economic situation (minimum wages*)	
1 to 3	18(90.0)
4 to 6	2(10.0)
Marital status	
Married	13(65.0)
Common-law marriage	5(25.0)
Single	2(10.0)

* Value of minimum wage in 2016: R$ 880,00.

Their diagnosis was varied, 4 women with condylomatosis (20.0%), 5 women with grade II cervical intraepithelial neoplasia (CIN) (25.0%), 7 with grade III CIN (35.0%), 2 with vulvar intraepithelial neoplasia (10.0%), and 2 with intraepithelial vaginal neoplasia (10.0%). From the data analysis, three thematic units emerged: physical and emotional changes; changes in sexual and affective relationships; changes in social relationships.

### Physical and Emotional Changes

The women, as participants in the study, reported physical and emotional changes in their lives related to HPV infection. In the physical changes, abdominal pain (cramps) and genitalia pain were evidenced.

[…] I feel pain when I clean the house. Sometimes, when I make some effort at home I feel cramps (E2).

I have pain in my “lower stomach”and when I pee (E3).

[…] I feel pain in my belly […] (E15).

I feel pain when I wash it (genitalia) in the shower (E19).

Pain is perceived while carrying out everyday activities such as housekeeping and showering. Also, the presence of pruritus (itching) was also found in the speeches of the participants.

I feel itchy a lot […] (E8)

[…] sometimes I feel itchy (E15).

I feel itchy a lot […] it bothers me when I sit down […] (E19).

Regarding the emotional changes, the testimonies of the women showed the feelings of worry, sadness, and despair regarding the diagnosis of HPV, especially when it has been discovered.

[…] it seems that just thinking about “this thing” makes me worried, sad […] (E7).

The day I found out, I only cried, I became very sad and worried […] (E8).

I was very sad when I found out, but nowadays, I don't keep thinking about it in order not to suffer […] (E9).

[…] when I discovered it, I was desperate […] (E13).

[…] I was very worried and a little desperate, I feel very ashamed […] (E15).

There is also a feeling of shame in relation to other people in their social life, such as their children, as they have an STI.

[…] I am very ashamed […] (E3).

[…] I got really bad when I found out. I kept thinking about what people would think of me (E16).

After I discovered it, I didn't even leave the house anymore because I thought people knew what I had […] (E19).

When I found out, I was “stuck.” I thought of my children. I was ashamed […] (E20).

With emotional changes, fear became present in the life of these women with HPV, through the knowledge of the association of the virus with the risk of cancer and, consequently, the fear of death.

When I discovered it, I was very scared of cancer and I was thinking that my hair would fall out and I would get thin […] (E2).

[…] I thought it was very serious, I thought it was cancer and I was afraid of dying (E10).

[…] I'm very scared of dying because of this… I can't die because I have two daughters to raise (E1).

### Changes in Sexual and Affective Relationships

Changes in the sexual and affective relationships of the women related to HPV were reported. The speeches show the loss of desire of the women and feeling of embarrassment of having sex after the HPV diagnosis.

[…] I have less desire to have sex […] (E1).

[…] I have almost no intercourse with my husband because I no longer have any desire (E8).

[…] now I have less desire […] (E15).

At the beginning of the treatment, I had no desire to have intercourse […] (E16).

At first, I felt very ashamed and didn't feel like doing anything, and it continued and we finished […] (E19).

The sensation and fear of pain, discomfort, and bleeding during sexual intercourse was reported several times, also negatively influencing having sex.

[…] I'm afraid of it hurting. Because sometimes, depending on the position, it hurts. I feel a lot of pain during sex […] (E2).

[…] In the beginning, it hurt a lot to have intercourse. […] I feel pain during intercourse, and it bothers me (E3).

[…] I only feel pain when I have sex […] (E7).

[…] I was afraid of it hurting […] (E10).

[…] sometimes, it bleeds during sex […] (E11).

[…] sometimes, when I have intercourse, it bleeds […] (E15).

Interruption of sexual activities correlating with HPV infection was also evidenced.

[…] Since I discovered that I had this, 4 years ago, I have not had sex again (E6).

[…] After the doctor discovered it here, I did not have sex until the day I had the laser (E12).

Also, as a consequence of the changes in sexual life, conjugal problems emerged. Dyspareunia and decreased libido are corroborating conditions for this situation.

[…] My husband always complains because I feel a lot of pain […] (E1).

I almost have no intercourse with my husband anymore because I don't want to, and he always gets angry about it […] (E8).

The risk of infidelity and the mistrust of the partner were also highlighted by several women as a conflictive situation, causing wear and tear in the affective relationship.

[…] I get angry, you know? Because my husband always had other women, and he never caught anything! “It” never appeared in him […] (E1).

[…] I don't trust my partner, and I have already quarreled a lot with him […] (E5).

[…] I was very suspicious of my boyfriend, but he said it was not him because he had nothing […] (E11).

[…] at first, I mistrusted him […] (E17).

It is also possible to verify that condom use encouraged by the partner and the fear of infertility also affected the affective relationships.

[…] I never used condoms because my husband never let me (E2).

[…] When I told my husband, he didn't like it because he was afraid that I couldn't have any more children (E3).

On the other hand, there were three partners (15%) who faced the situation in a positive way, offering support and strengthening the affective relationship.

[…] in the beginning, my husband thought that I didn't want to have intercourse, but now he understands the situation […] (E10).

[…] I told my husband and he faced it very well […] (E13).

The relationship between me and my husband remains the same […] (E15).

### Changes in the Social Relationships of Women with HPV Infection

The changes in social relationships included two antagonistic situations. Most of the interviewees affirmed that they maintained normal social activities, even with HPV infection.

[…] I don't stop going out because of this disease […] (E2).

[…] no, nothing has changed in my daily life […] (E4).

However, some women reported lack of motivation to enjoy social activities after the diagnosis of HPV.

[…] after I found out (HPV), I didn't even leave the house because I thought people knew what I had […] (E19).

Also, HPV infection and its treatment had an impact on women's professional lives, mainly in terms of work-related absenteeism, leading in some cases to them losing their job.

[…] I always miss work so I came here […] (E3).

[…] I have had to miss my work on many occasions to come here (E6).

[…] I lost my job because I always needed to be absent so I came here (E20).

To face this difficulty, participant E10 reported the strategy used to not have a change in professional life.

[…] There was no change in what I do because I work (I'm a cleaner), but I come here the day I can be absent from my work […] (E10).

## Discussion

When women face the fact that they have HPV infection, they go through different situations that change their daily life by having to face the peculiarities of the diagnosis and to adapt to this new reality.^17^ ^18^ Such a condition leads to physical, emotional, affective, sexual, and social changes.

In the present study, it was possible to verify physical changes such as pain and pruritus caused by HPV infection. However, most HPV infections are asymptomatic or not apparent and are transient, and may spontaneously disappear. It is estimated that in only 5% of infected people will there be some form of manifestation. However, as the disease progresses, precursor lesions or early-stage cancer may result in vaginal bleeding, discharge, and pain.^19^

The emotional aspect was also affected as a result of the infection caused by HPV. The positive diagnosis of an STI brings about feelings such as sadness, confusion, fear, fear of being judged, malaise, family contempt, partner anger, and bargaining behavior.^20^

Corroborating the results found in the present study, an investigation performed to assess the psychosocial burden and impact on the quality of life of HPV-related diseases observed a significant psychosocial impact in women.^21^ Regarding the feelings of shame described by some women, Yang et al^22^ reported that the impact of shame and stigma on the diagnosis of HPV disclosure may influence the initial decision to use the vaccine.

In a study designed to assess the prevalence of shame in women diagnosed with HPV/CIN, these women had a substantially higher state of shame than women diagnosed with another STI.^23^

Concerning feelings of fear of cancer and death, similar findings have been reported in studies involving women with HPV.^24^

In terms of sexual and affective aspects, the study by Dominiak-Felden et al^21^ determined that infections caused by HPV have a negative impact on sexual functioning, especially in those diagnosed with CIN II and/or CIN III when compared with women in the general population.

Corroborating with the findings, a study conducted in Fortaleza, state of Ceará, Brazil, with 100 women diagnosed with HPV infection showed that 55.5% of the women stated there was a decrease in libido; also, 60.0% reported a decrease in the frequency of intercourse, and 37.8% reported sexual abstinence.^25^

Regarding the complaints of dyspareunia and leucorrhea, an investigation performed in Ecuador found an association between exposure to HPV infection and the presence of whitish vaginal secretion and dyspareunia.^26^

Thus, one of the most significant characteristics found in women diagnosed with HPV is the genital change, which may not only cause pain and bleeding in sexual intercourse but also shame and embarrassment, which could lead to marital problems.^17^ ^27^ ^28^

Thus, legitimating the findings of the present study, the distrust and the risk of infidelity were also pointed out by Jeng et al^29^ as possible causes of conflict with the partner because they believed that alleged extramarital affairs could be the source of the infection.

In terms of the non-use of condoms, a similar result was found in a Brazilian study involving 977 women diagnosed with HPV infection in which it was possible to verify that the non-use of protection methods in stable relationships suggests an excessive confidence in the affective partner.^7^

However, the risk of cervical cancer and the fear of infertility are real concerns, especially in the lives of women with HPV infection, as described in a multicenter study in China.^28^

Despite the strengthening of affective-sexual relationships being a threat to the use of protection methods, these relationships are an important source of emotional support when there are fears of infection, and they also give practical support, helping in domestic activities or accompanying the patient to medical consultations.^27^

Regarding the social relationships changes, although most of the participants in the present study have normal social activities, and the lack of motivation to enjoy social activities and absenteeism at work with risk of layoffs are reported, corroborating with other studies.^28^ ^30^ ^31^

The HPV-related stigma added to misinformation leads to isolation through fear of disclosure of the diagnosis and negative self-image compared with other people.^31^

Regarding the social changes, similar aspects were revealed in a study developed in Ethiopia, in which the number of days of being absent from work for outpatient follow-up or hospitalization were quantified, with frequent absenteeism threatening the maintenance of employment.^30^

In this sense, considering the changes related to living with HPV infection, the importance of support networks can be seen. A study performed in Mexico pointed out that informal networks such as family and friends, and formal networks such as churches, support groups, and medical professionals and their guidelines help people to cope with HPV infection.^27^ Thus, the emotional burden is inversely proportional to the knowledge of the infection.^28^ ^31^

The present study allows changes in the quality of life of women with HPV infection to be seen and, therefore, enables the management of and intervention in situations that can significantly affect treatment.

## Conclusion

The results show that, in general, women with HPV infection face a variety of physical, emotional, affective-sexual, and social changes, modifying their quality of life. Complaints of pain, pruritus and the presence of warts, discharge and bleeding can decrease libido and negatively impact self-image, influencing affective-sexual relationships. Human papillomavirus-related stigma and shame at the diagnosis lead to the loss of motivation of social activities, and absences from work for outpatient follow-up increase the fear of disclosure, as well as being a risk for future dismissals. Therefore, HPV infection can lead to exponential changes in the quality of life of women and can be mitigated by the availability of sources of support such as family, friends and the multi-professional team, helping to improve knowledge and cope with HPV.
